# Evaluating Research Centers in Minority Institutions: Framework, Metrics, Best Practices, and Challenges

**DOI:** 10.3390/ijerph17228373

**Published:** 2020-11-12

**Authors:** Angela Sy, Traci Hayes, Kelly Laurila, Carlamarie Noboa, Robbert J. Langwerden, Michelle M. Hospital, Doris A. Andújar-Pérez, Lakesha Stevenson, Suzanne M. Randolph Cunningham, Latrice Rollins, Hala Madanat, Tanya Penn, Shiva Mehravaran

**Affiliations:** 1 Department of Tropical Medicine, Medical Microbiology, and Pharmacology, John A. Burns School of Medicine, University of Hawaii, Honolulu, HI 96813, USA; sya@hawaii.edu; 2 Department of Public Health, School of Health Professions, University of Southern Mississippi, Hattiesburg, MS 39406, USA; 3 Department of Anthropology, College of Social and Behavioral Sciences, Northern Arizona University, Flagstaff, AZ 86011, USA; kelly.laurila@nau.edu; 4 Department of Surgical Sciences, School of Dental Medicine, University of Puerto Rico, Medical Sciences Campus, 00938 San Juan, Puerto Rico; cmnoboaramos@gmail.com; 5 Community-Based Research Institute, Florida International University, Miami, FL 33199, USA; rlangwer@fiu.edu; 6 Department of Biostatistics, Robert Stempel College of Public Health & Social Work, Florida International University, Miami, FL 33199, USA; hospitam@fiu.edu; 7 Ponce Research Institute, Ponce Health Sciences University, Ponce 00732, Puerto Rico; dandujar@psm.edu; 8 Center for Cancer Research and Therapeutic Development, Clark Atlanta University, Atlanta, GA 30314, USA; stevensonevaluation@gmail.com; 9 Center for Community Prevention and Treatment Research, Division of Research and Evaluation, The MayaTech Corporation, Silver Spring, MD 20910, USA; srandolph@mayatech.com; 10 Department of Community Health and Preventive Medicine, Morehouse School of Medicine, Atlanta, GA 30310, USA; lrollins@msm.edu; 11 Division of Research and Innovation, San Diego State University, San Diego, CA 92182, USA; hmadanat@sdsu.edu; 12 Institute of Public Health, School of Public Health, San Diego State University, San Diego, CA 92182, USA; tpenn@sdsu.edu; 13 Department of Computer Science, School of Computer, Mathematical, and Natural Sciences, Morgan State University, Baltimore, MD 21251, USA; shiva.mehravaran@morgan.edu

**Keywords:** RCMI, program evaluation, evaluation metrics, collaboration, consortium-wide evaluation, evaluation framework, health disparities

## Abstract

The NIH-funded Research Centers in Minority Institutions (RCMI) program is currently funding 18 academic institutions to strengthen the research environment and contribution to health disparities research. The purpose of this multiphase mixed-methods study was to establish a uniform evaluation framework for demonstrating the collective success of this research consortium. Methods included discussions of aims and logic models at the RCMI Evaluators’ Workshop, a literature review to inform an evaluation conceptual framework, and a case study survey to obtain evaluation-related information and metrics. Ten RCMIs participated in the workshop and 14 submitted responses to the survey. The resultant RCMI Evaluation Conceptual Model presents a practical ongoing approach to document RCMIs’ impacts on health disparities. Survey results identified 37 common metrics under four primary categories. Evaluation challenges were issues related to limited human resources, data collection, decision-making, defining metrics, cost-sharing, and revenue-generation. There is a need for further collaborative efforts across RCMI sites to engage program leadership and community stakeholders in addressing the identified evaluation challenges and measurement. Program leadership should be engaged to apply the Evaluation Conceptual Framework and common metrics to allow for valid inter-institutional comparisons and consortium-wide evaluations. Stakeholders could ensure evaluation metrics are used to facilitate community impacts.

## 1. Introduction

The National Institutes of Health (NIH) has various types of grants to support research- related programs and funds over 1200 research centers at $41.7 billion USD annually [1]. The NIH expects these research centers to demonstrate how they ultimately contribute to impactful science [2,3]. Ideally, research centers demonstrate both an ‘academic impact’ of fostering research, with a ‘wider and societal impact’ that includes benefits outside of academia, e.g., for communities [2]. NIH research centers are funded with collective goals to decrease morbidity and mortality [4]. Strong program and system-level evaluations of research centers are essential to guide and improve program progress and demonstrate successes [5].

### 1.1. Evaluation of Academic Research Centers

The links between scientific research, practice, and impact to community and society can be complex. This is especially true for research centers, which are funded to be multifaceted. These centers often involve a variety of investigators from different backgrounds and training to collaborate on research projects that are aligned with the goals of the academic research center [5,6] and the mission of the funder.

Evaluation that focuses on research projects’ outcomes and impacts can often take years after funding to establish the value of the research center conducting the research project, while neglecting to demonstrate other key accomplishments of the funded activities. Given the complexity of what (and how) research efforts are translated to the community, funders should acknowledge the approaches that allow evaluation of earlier phases of research and research centers’ program activities. A focus on a variety of process measures is critical for demonstrating the broad range of impact of research centers [2,6]. The focus on processes, along with outcomes and impacts when available, provides insight on how an academic research center may influence research activities, starting at the level of investigators directly involved in the center [7].

### 1.2. Research Centers in Minority Institutions

The Research Centers in Minority Institutions (RCMI) program was initiated in 1985 by the NIH to strengthen the research environment and participation of minority serving institutions and increase resources for health disparities research. The program is a U54 award mechanism administered by the NIH National Institute on Minority Health and Health Disparities (NIMHD) and provides support to specialized research centers that have historically trained and awarded doctorate degrees in health professions and health-related sciences to individuals from underrepresented, underserved, and minority populations. The 18 currently active RCMI sites, which are funded under three Requests for Applications (RFA-MD-18-012, RFA-MD-17-006, and RFA-MD-17-003), are listed in Appendix A. All RCMI awards are competitively funded to support infrastructure and research development [8]. RCMIs represent a variety of academic institutions that serve diverse geographic, ethnic-racial, and socio-economic communities [9].

The RCMI programs’ national goals are to: (1) enhance institutional research capacity within the areas of basic biomedical, behavioral and/or clinical research; (2) enable all levels of investigators to become more successful in obtaining competitive extramural support, especially from NIH, particularly on diseases that disproportionately affect minority and other health disparity populations; (3) foster environments conducive to career enhancement with a special emphasis on development of new and early career investigators; (4) enhance the quality of all scientific inquiry and promote research on minority health and health disparities; and (5) establish sustainable relationships with community-based organizations that will partner with the RCMI site.

As academic research centers sponsored by NIH, the overall RCMI program addresses common goals of other research support mechanisms while having unique objectives for each RCMI outlined in goal (4) and goal (5) mentioned above. Community participation in decision-making processes is essential to ensure the proper tailoring of research toward the respective communities [10]. Therefore, since 2016, RCMI Funding Opportunity Announcements have required specific community engagement components—goal (5); to address health disparities through the research effort—goal (4).

The RCMIs vary in purpose, core components, size, and infrastructure, but all are required to evaluate the impact of the center. According to NIMHD [9], the proposed activities, cores, and projects must be evaluated to determine impact including its ability to enhance institutional research infrastructure and increase the scientific productivity of investigators. A solid evaluation framework can increase the likelihood of research center programs achieving their goals and objectives, thus, ensuring the sustainability and viability of the award mechanism [7].

### 1.3. The Need for a Uniform RCMI Evaluation Framework

Since 2007, the RCMIs have worked collaboratively to centralize their tools and applications to improve evaluation tracking [8]. An evaluation framework not only aids each RCMI in assessing the achievement of the program’s goals and objectives, which is essential to its viability and sustainability, but allows standardization and/or harmonization of common evaluation metrics across the centers. A well-devised evaluation model can improve program planning and development, as well as clarify programmatic goals and objectives [11,12,13,14].

Evaluation involves applying methodical approaches that are valid and reliable, and arriving at findings that can influence program services and outcomes [9]. The evaluative process for obtaining quality data and metrics for strategic planning and decision-making is increasingly recognized in the area of health disparities [15]. Determining whether the program is positioned to ensure the metrics are achieved requires obtaining rich data from internal and external sources [8]. The data can inform sequential components of the logic model (the inputs, activities, outputs, and impacts) of the RCMI and can be examined to help determine performance and outcomes related to the mission [11]. For example, increases in scientific collaborations benefit research productivity [16] and may eliminate barriers for early career investigators [17]. Not only may collaboration lead to increased scientific productivity, but also—and arguably more importantly—these inputs lead to expanded professional networks, potential access to a larger variety of expert mentors for investigators, and creation of other collaboration (research infrastructure) opportunities that may have not arisen otherwise [17]. Impacts can include the level of satisfaction expressed by program officers and outcomes associated with community uptake of research findings [11].

Evaluating programs aimed at addressing health disparities provides attention to specific elements such as population characteristics and the role of social determinants of health [15]. Accordingly, the RCMI evaluation effort requires a particular focus on identifying and demonstrating the added value of community-engaged research to address and reduce health disparities. A 2013 Institute of Medicine report recommended that academic institutions funded by the NIH Clinical and Translational Science Awards (CTSA) consortium engage communities across the full spectrum of translational research. In an assessment of community engagement in CTSAs research, institutions reported that unique institutional priorities created barriers to developing shared metrics. The assessment also found an overall lack of attention among the CTSA consortium to develop and deploy metrics to assess community engagement in and contributions to research. As a result, the assessment recommended that defining and measuring community engagement within translational science requires increased institutional commitment [18]. Another example is the NIH-sponsored National Cancer Institute’s Partnerships to Advance Cancer Health Equities (PACHE) initiative that builds institutional research capacity to reduce health disparities or advance health equities. PACHE program evaluation is focused primarily on designs for complex, community-engaged research partnerships [19]; evidence to support how infrastructure programs increase research capacity among underrepresented investigators [20]; strategies for expansion of the pool of health disparity researchers [21]; evaluation of underrepresented focused health disparities research training programs serving as a pipeline to build capacity for underrepresented investigators and reduce health disparities [22]; and strategies for building research capacity among faculty [23].

To address the need for a uniform evaluation framework, we designed this multiphase mixed-methods collaborative study to (a) develop an RCMI evaluation conceptual framework; (b) identify a comprehensive set of shared metrics; and (c) discuss challenges and best practices for evaluating the RCMI programs. Applying this framework across the RCMI consortium will facilitate demonstrating the short- and long-term success of the collective efforts of the awarded RCMIs and allow for valid comparisons in identifying effective interventions. For the purpose of this paper, the terms “framework” and “model” are used interchangeably throughout the manuscript.

## 2. Materials and Methods

Information to develop an evaluation conceptual model to frame and identify common metrics, best practices, and challenges was obtained using a multiphase mixed-methods approach: an in-depth discussion at a workshop for RCMI evaluators, synthesis of logic model metrics to identify common metrics, iterative discussions about a proposed conceptual framework and a common metrics survey questionnaire administered to RCMIs. All RCMIs’ evaluators and/or other key individuals were invited to participate in data collection activities: the workshop, iterative discussions, and the common metrics survey.

### 2.1. Phase 1: RCMI Evaluators’ Workshop (December 2019)

The evaluation experts from each of the RCMI were invited to participate in a discussion workshop of RCMI evaluation methods, indicators, and metrics at the RCMI 2019 National Conference, Collaborative Solutions to Improve Minority Health and Reduce Health Disparities, held December 14 and 15, in Bethesda, Maryland. The RCMI conference hosts emailed invitations directly to RCMI evaluators. Initially, the RCMI conference hosts included this “by invitation only” session for RCMI evaluators and/or designees as part of the conference registration process. Later the workshop was open to any conference attendees.

The purpose of the workshop was to elucidate RCMIs evaluation challenges and successes and to establish a comprehensive evaluation framework that includes how community engagement may contribute to reduction in health disparities. The workshop was facilitated by two representatives of the RCMI Translational Research Network (A.S. and T.H.) and two RCMI evaluators (K.L. and L.R.). At the workshop, the participants discussed a comprehensive evaluation framework that establishes standards and priorities for a synergistic consortium while considering the unique needs of the individual RCMIs. The evaluators were asked to prepare materials (logic model, short/medium term goals for each core, barriers, challenges, and best practices) in advance of the workshop as a tool to engage in meaningful discussion. Each evaluator in attendance shared this information in individual presentations and made the information available to the entire group via a shared “cloud based” folder.

### 2.2. Phase 2: Developing the RCMI Conceptual Evaluation Model (January 2020)

After the workshop, a report summarizing key points was disseminated to participants to guide subsequent discussions for the evaluators working group (EWG) and develop the RCMI evaluation framework. The RCMI evaluators were invited to participate in two meetings to identify next steps for developing the framework. Collaborating via bimonthly videoconferences, the EWG synthesized data from workshop presentation slides and other materials prepared by each participating RCMI that included program logic models and existing evaluation frameworks. The EWG also conducted a literature review of existing evaluation frameworks and conceptual models to inform the development of the proposed evaluation model [24,25]. Further, the EWG defined components of a conceptual model. Using the iterative nature of this multiphase mixed-methods process, information gleaned in subsequent phases was also used to inform the final conceptual model.

### 2.3. Phase 3: Identifying Common and Key Evaluation Metrics (January–February 2020)

To develop a metrics table that was common across sites, the EWG requested a copy of logic models from participating evaluators (*n* = 10); eight sites shared a logic model. The metrics were methodically selected from logic models and classified as primary targets, secondary targets, outcomes, and impact metrics. Metrics were also examined based on their recurrence in the logic models. Less common metrics were included in the model with an asterisk. Next, the EWG reviewed the metrics and excluded those that were deemed irrelevant based on general consensus.

### 2.4. Phase 4: A Case Study: (March–June 2020)

The EWG developed a survey questionnaire based on the key metrics identified in previous phases to outline definitions, approaches, and data collection practices used among current RCMI evaluators. Evaluators of currently funded RCMIs (*n* = 18) were invited via email to complete the online survey with reminders. Six key metrics associated with primary evaluation targets were selected. The survey contained eight questions about how each of the metrics are conceptualized and measured. The final instrument consisted of 62 questions, including a section (12 questions) related to the RCMI site information. For the purpose of this paper the EWG focused on finding answers to the following key questions:

1. How was the metric operationalized?
2. What are the primary approaches and methods for data collection?
3. Does the RCMI use primary or secondary data?
4. What is the periodicity for data collection?

In the analysis of open-ended responses to the survey items, a thematic coding process was used to identify common themes and outliers. Two EWG members examined responses to each question and identified themes that emerged from their independent coding process. After independent coding, the evaluators met and iteratively refined the themes to verify inter-rater reliability. Once the process was complete, frequencies and percent of respondents who mentioned each of the themes were calculated.

## 3. Results

### 3.1. Phase 1: RCMI Evaluators’ Workshop

Nineteen RCMI evaluation representatives (i.e., evaluators, principal investigators—PIs, and other key program staff) representing 10 RCMIs attended the facilitated workshop. The discussion explored in-depth, the programs’ various metrics and measures, the challenges of evaluating the multi-faceted programs, and the resources utilized and needed to collect and manage the data to address key performance measures.

Evaluation best practices and challenges shared during the RCMI Evaluator’s Workshop included a focus on the importance of (1) continuous RCMI team/institutional engagement, (2) establishing a multidisciplinary evaluation team, and (3) extracting multiple data sources to obtain baseline data for ongoing annual benchmarking and systematic data collection. The challenges in conducting RCMI evaluations involved barriers in being able to conduct the evaluations and limited evaluation guidance comprised of (1) RCMI cost-sharing, (2) revenue generation through charge-back plans to collect fees from researchers funded by other grants who use RCMI-funded facilities/equipment, (3) data collection, access to the data and infrastructure, (4) decision making (about evaluation), (5) disaster/crisis management (e.g., financial, natural disasters), (6) having full time employees and staffing (FTEs), and (7) how to evaluate the broader community impact of the RCMI program. The RCMIs shared that they were diligent in their efforts to incorporate technologies to streamline data collection and improve monitoring. To improve evaluation tracking, the RCMI’s use online platforms (Altmerics, Scopus, NIH RePORTER, PubMed, etc.) that track scholarly activities and use diverse data management tools including Research Electronic Data Capture (REDCap, Vanderbilt University, Nashville, TN, USA), Profiles Networking (Harvard University, Cambridge, MA, USA) and eagle-I (Harvard University, Cambridge, MA, USA).

### 3.2. Phase 2: RCMI Evaluation Conceptual Model

**Figure 1:**
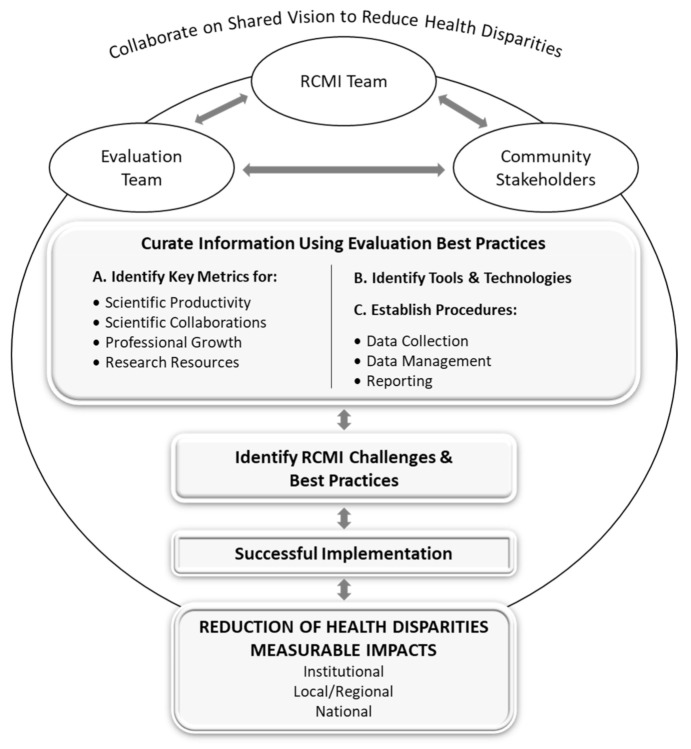
The Research Centers in Minority Institutions (RCMI) Evaluation Conceptual Model developed through an iterative process of in-depth discussions among RCMI evaluators, review of the literature, and results of the survey administered in this study.

The iterative process of the in-depth discussions among the EWG and review of the literature resulted in the RCMI Evaluation Conceptual Model and the identification of key evaluation metrics and extrapolated measures (Figure 1). The model was then further refined using information collected in Phase 4 of the study. The proposed model emphasizes a practical on-going evaluation approach to document the RCMIs’ institutional and national impact consistent with NIMHD’s mission to address health disparities. It summarizes essential elements of the program and system-level evaluation and seeks to present commonalities and variances that affect program effectiveness. The model also considers the complex and multi-dimensional nature of research on minority health and the reduction of health disparities, including research that crosses domains and levels of influence [9]. A detailed description on how the RCMI Evaluation Conceptual Model will guide evaluation efforts of RCMIs (framed with the literature) is provided.

The model reflects how the RCMI programs share a common vision that aligns with that of the NIMHD. The RCMI programs individually and collectively aim to reduce health disparities among minority and other underserved populations through collaborative, interdisciplinary, transdisciplinary, and community engaged research. Community impacts contribute to national efforts to reduce health disparities through collaborative translational research.

The RCMI evaluation teams are the curators of the site metrics and direct the evaluation of the program performance, coordinate and harmonize data collection, and enable the integration of relevant components for favorable program amendment [26]. The evaluation team works with all stakeholders to ensure documentation and records are maintained and available for ongoing program monitoring. The RCMI program administration works in tandem with the evaluation team and the community stakeholders, who consist of professionally, geographically, and socio-demographically diverse individuals representing academia, industry, community partners, and government interested in discovery and implementation [13]. The stakeholders are partners in the evaluation process contributing information and knowledge to obtain greater capacity to achieve the shared vision and program goal. Meaningful collaboration at various programmatic levels is fundamental to the RCMI program and is essential for transdisciplinary research [13].

Key metrics are performance measures critical in determining the effectiveness and efficiency of the program toward achieving its mission [11]. Universal success metrics of research infrastructure programs include the number of publications acknowledging the program funder and grant number, researchers hired to the program, and grants submitted and awarded to faculty funded by the RCMI. Other metrics are novel and aim to evaluate the conceptual outcomes such as, “increased trust in the research process” and “increased willingness to access local health services.” The value of these factors varied and, therefore, the inclusion of a factor in a specific metric category also varied. Most importantly, the RCMI key metrics were broadly categorized into: (1) scientific productivity, (2) scientific collaborations, (3) professional growth, and (4) research resources. Although establishing common metrics for the reduction of health disparities was beyond the scope of this study, future collaborations should work to establish these.

Tools and technologies contribute to the collaborative process of the RCMI programs. The management of evaluation data requires the use of widely accepted applications and those designed for specific site needs. The evaluation process relies on data curation tools and analytical approaches to document the integration of the various program components and core facilities (cores). Widely accepted tools such as the NIH RePORTER (https://reporter.nih.gov/) or Altmetric (Altmetric, London, UK), and customizable applications such as Profiles Networking (Harvard University, Cambridge, MA, USA), along with quantitative (e.g., REDCap) and qualitative data collection analysis tools facilitate a thorough and efficient evaluation process [8]. The curation of the types of data varies by institution, but the requirement to show productivity and value-added of the RCMI program is universal.

### 3.3. Phase 3: RCMI Evaluation Metrics

In phase 3, the EWG reviewed information provided by RCMIs to identify common evaluation metrics from logic models shared by evaluation group representing eight RCMI evaluation groups. Table 1 summarizes common metrics used by RCMI evaluation teams that are used to evaluate the progress of RCMI efforts that aim to support and expand health disparities research. The table is organized in four parts encompassing broad primary and secondary targets along with examples of commonly used outcome metrics. On the broadest level (and reflected in the model described in the previous sections), four overarching essential areas (primary targets) of focus for evaluation commonalities across all RCMIs were identified—increasing scientific productivity, increasing scientific collaborations, fostering professional growth, and expanding research resources—and are described in detail.

First, increasing scientific productivity with regards to research focusing on health disparities and minority health was identified as a primary focus of the RCMIs as part of the NIMHD RCMI funding mechanism. This area is intertwined with the RCMI program’s goals of fostering health disparity research by growing infrastructures that enable collaborative investigative work. Secondary targets in this area provide metrics for the growth of scientific studies, defined by pilot project specific productivity, funded grants, publications, scientific dissemination, and community dissemination. Examples of outcome metrics include—(1) number of grant submissions, (2) number of grant awards, (3) number of peer-reviewed publications, and (4) number of presentations or symposia given (conference/symposia or community-based).

Second, increasing scientific collaborations is a broad primary target for measuring the growth of the RCMI research infrastructures. Secondary targets commonly utilized as indicators of research growth within an RCMI are evaluating both the number and types of research partners and community partners. Scientific collaborations are often measured as process metrics, such as networking events or outcome metrics, such as the number of grant collaborators.

An increasingly important component of the RCMI program is community engagement, as new and renewed RCMIs funded since 2016 are required to include a Community Engagement Core. Measures related to this concept include (1) number of memoranda of understanding (MOUs) signed, (2) expansion of community advisory boards, and (3) academic-community grants submitted.

The third primary target area for evaluating RCMI programs, fostering professional growth, focuses on the training, professional development, and support of early career investigators (ECIs), particularly those who are from historically underrepresented groups in academia (i.e., race, ethnicity, and gender). Comprehensive evaluation that entails both results on process and outcomes of various professional development efforts is important in the context of how RCMIs foster career development of RCMI-funded early investigators.

The fourth primary target area for RCMI evaluation, expanding research resources includes metrics that evaluate the growth in physical infrastructure, intellectual resources, and hiring faculty needed to conduct and expand health disparities and minority health research. Metrics in this category include the number of training seminars/workshops (e.g., statistics or research methodology) and/or biostatistics consultations for RCMI-affiliated faculty or early-career investigators. Other metrics in this area frequently include the tabulation of increases in physical research space, acquisition of technology or software, and developing or expanding data repository capacity. Finally, the number of researchers hired as a result of RCMI recruitment efforts is often documented as a direct measure of expansion of the RCMIs’ resources.

### 3.4. Phase 4: Case Study

Fourteen of the 18 RCMIs responded to the survey; only one set of responses was collected from each RCMI. Table 2 summarizes the number of responses to the case study survey. Response rates ranged from 55.6%, (*n* = 10) for scientific productivity (publications) and fostering professional growth (mentoring) metrics to 11.1% (*n* = 2) for the health disparity (external grants submitted) metric (Table 2). Select items that are non-identifying from the RCMI site profile are included in the findings, including from NIH RePORTER. The mean duration of funding of these RCMIs is 20 years (SD = 14.4) with nine that are over 27 years old.

The EWG compiled the case study results to discuss the breadth of approaches used to measure and operationalize the same metrics across centers. The case study survey results are summarized in Table 3, Table 4, Table 5, Table 6 and Table 7. The findings related to the metric selected for each primary evaluation target included in the survey are discussed including how metrics are operationalized and measured across RCMIs. The number of responses for each sub-question may be greater than the number of respondents, because sub-questions were coded with multiple themes.

#### 3.4.1. Scientific Productivity

The metric, number of peer-reviewed publications, was selected from the measures for scientific productivity (primary target). As presented in Table 3, all nine RCMIs which responded to this sub-question operationalized the metric as publications in a peer-reviewed journal by investigators, studies, or affiliated faculty supported by RCMI. Progress reports/surveys and online databases, such as Scopus, Google Scholar, Web of Science, among others were reported by eight of ten RCMIs. All nine responding institutions indicated that they collected data from both primary and secondary sources. Five of nine responses each indicated data were collected at variable time points (annually, bi-annually, monthly, or bi-monthly).

#### 3.4.2. Scientific Collaboration

A measure of scientific collaboration examined was the number of research partners (Table 4). Eight of the nine respondents operationalized the metric as an individual or group participating in grant/research related activities (e.g., mentors, PI, co-investigator/s), organization, consortium, etc.). Four of eight responses indicated survey and tracking system/administrative records as the most common data collection method. All eight responding institutions reported that they collected data from both primary and secondary sources. Three of eight responses indicated that data collection was ongoing, biannual, and annual.

#### 3.4.3. Professional Growth

Mentoring quality was examined as a metric for fostering the *professional growth* of underrepresented investigators, a secondary target (Table 5). Eight of nine respondents operationalized this metric as the perceived quality and satisfaction of professional growth activities for underrepresented investigators. All eight respondents indicated using survey methods to collect mentoring quality data. Six of nine respondents reported both primary and secondary data sources of collection. Data collection is conducted annually.

#### 3.4.4. Research Resources

Intellectual resources was a secondary target measured as the number of biostatistics consults, workshops, seminars, and training (Table 6). All seven respondents operationalized the metric as training activities (workshops, seminars) offered through RCMI communities. All eight respondents indicated data collection occurs through questionnaires or surveys with seven of eight respondents reporting primary data collection. Seven of eight responding RCMIs reported ongoing data collection efforts.

#### 3.4.5. Community Engagement

A metric of community engagement examined was the number of formal agreements, MOUs or partnerships with community partners (Table 7). Five of six respondents operationalized the measure as (1) community members are involved in the RCMI activities and decision making, and (2) community partnerships are included in proposals and research studies supported by RCMI. Six of seven institutions responded that surveys and interviews were used to collect data. Four of seven respondents indicated that data were collected from both primary and secondary sources. All six responding institutions indicated that data collection for their community engagement measure was ongoing.

#### 3.4.6. Health Disparities

The number of external health disparity focused grants submitted by RCMI-funded pilot project investigators was the metric selected to measure health disparities. Given the very low response rate (11.1%, *n* = 2), results are not reported.

## 4. Discussion

The aims of this multiphase mixed-methods RCMI evaluation study were threefold: (1) develop an RCMI conceptual evaluation framework; (2) identify and examine shared evaluation metrics; and (3) identify and discuss challenges and best practices for evaluating the RCMI programs. The multiphase study resulted in establishing a uniform evaluation framework and specific metrics that can be used to demonstrate short and long-term success of the individual RCMIs and the collective RCMI consortium.

The RCMI Evaluation Conceptual Model (Figure 1) illustrates the importance of collaborations with the key stakeholders (evaluation team, RCMI team, and other stakeholders) within each RCMI for a shared vision on evaluation approaches, activities, and procedures of each RCMI toward reducing health disparities. Similar to other research infrastructure programs that have addressed the need for common metrics (e.g., the CTSA) [7], the subsequent key metrics identified in this study focus on stimulating and expanding research capacity for the RCMI. Moreover, integrating the evaluation tools and technologies, as well as best practices and procedures to collect, manage, and report the findings were identified as an essential part of the evaluation framework.

Previous examinations of research center evaluation metrics noted that in addition to those commonly used for academic research centers, expanded indicators are needed to sufficiently address the complexity of research initiatives such as the RCMI [27]. To that end, in this project, the RCMI Evaluation Conceptual Model includes another essential aspect of a thriving evaluation process—i.e., identifying and addressing ongoing program challenges. In order to maximize the usefulness of the evaluation, the feedback process should be iterative, flexible, and dynamic. Incorporating these distinctions from its internal and external stakeholders ensures the RCMIs are adaptable and capable of reasonably responding to everchanging issues of the health environment while providing relevant research products [28]. The evaluation team should continuously document programmatic challenges (“what can the RCMI improve?”) and best practices (“what is the RCMI doing that is highly successful or innovative?”); and engage with the RCMI leadership and community stakeholders to share those findings so they can make data-driven decisions that inform program improvement. The RCMI leadership must be adaptable and open to implementing changes. For instance, the current global pandemic has exposed us to a “new normal” and resulted in a transition to and adaptation of new technologies that warrants new metrics. Successful uptake of evaluation feedback at the RCMI program level results in key outcomes for RCMIs: increased underrepresented investigator-generated research and institutional research capacity, strengthened research infrastructure, and established sustainable, community-engaged partnerships focused on RCMIs’ long-term aims to reduce health disparities.

Comprehensive metrics were identified for each of the primary and secondary evaluation targets. For each of the four primary target areas—scientific productivity, scientific collaborations, professional growth, and research resources—a key metric was selected for further examination to obtain greater detail on how these outcomes are measured and collected across RCMIs. Although all respondents reported utilizing both primary and secondary sources to measure scientific productivity, significant variability in secondary data sources was noted across RCMI programs. Since most of the secondary sources for publication data are open-source, the sponsor NIMHD could consider establishing standard metrics and practices of data abstraction about peer-reviewed publications among the tools available: Scopus, Google Scholar, Web of Science, PubMed, or PubCrawler.

Increases in scientific collaborations have been shown to benefit research productivity [16] and may eliminate barriers toward advancement for early career investigators [17]. There were diverse operational definitions to evaluate scientific collaborations (e.g., number of research partners). This is likely due to the variability of program composition nationwide, in terms of populations served, age of the RCMI program (number of years funded), and the configuration of the program cores. Several newer RCMIs reported offering community support (funding) for linking community and RCMI partners to facilitate partnership building, planting the seeds for future research partnerships. The variety of these measures demonstrates the sharing between RCMIs of evaluation ideas to benefit the overall RCMI evaluation effort. In addition to the common measure (number of collaborations), scientific collaborations could also be measured by the breadth of affiliations and locations of collaborators to include those within and outside of the institution, including scientific collaborations that are regional as well as national.

Fostering professional growth through quality mentoring of early career and underrepresented investigators was operationalized as the perceived quality and satisfaction of professional growth activities, mentorship benefit or impact, trainee needs, and promotion and tenure. Although the field has made some progress in this area [29,30], an ongoing RCMI evaluation challenge for this measure is that common tools and instruments to measure each of these constructs are not being used, limiting the capacity to make cross-site comparisons. Metrics for the research resources target area were the number of biostatistics consults/supports, workshops, seminars, and trainings. Institutions that responded reported collecting data for these metrics through surveys or questionnaires, and tracking systems and administrative records were widely used.

Community engagement (the number of formal agreements, MOUs, or partnerships with community partners) was operationalized as community members involved in the RCMI activities and decision making, formal community partnerships in proposals, and partners associated with research studies supported by RCMI-related/affiliated activities. Data sources for this metric varied widely, including surveys, interviews, tracking systems, administrative records, needs assessments, progress reports, and focus groups. In order to support a robust national evaluation of the RCMI program by the NIMHD, Centers could benefit from sharing specific tools and instruments for evaluating community engagement and reviewing them to find common questions. A review of community engagement metrics from similarly complex initiatives [31] and other common RCMI evaluation outcomes may also inform metrics for community engagement. For example the RCMI target “increase scientific collaboration” is also measured by “community partnerships.” Thus, those metrics that inform more than one RCMI target should be especially retained as they are robust evaluation measures.

Metrics and methods for data collection were not established for health disparities (e.g., the number of external health disparity-focused grants submitted by RCMI pilot project investigators) due to the low response on this outcome. In the RCMI context, reducing health disparities is a key dimension of the evaluation targets, not a stand-alone metric. Several examples to evaluate this outcome include grant proposals submitted with health disparity topics through the Investigator Development Core or an increase in underrepresented investigators at the RCMI institution who are conducting health disparities research. Additional metrics identified to evaluate health disparities as a target area would inform the RCMI impact and value added of this unique but necessary feature of RCMIs. At a minimum, a common understanding (shared vision) is needed among RCMI leadership, evaluators, and community stakeholders as to the interpretation of (what is meant by) this metric. Moreover, an agreement on operational definitions of key collective impacts for RCMI aligned with NIMHD’s mission such as “health disparity” and “health disparities research” as well as related concepts, such as “health inequities” and “social determinants of health”, would provide a shared vision across programs.

### 4.1. Recommendations and Future Directions

A well-defined evaluation approach is critical to RCMI program success. An inclusive pragmatic conceptual framework is an evaluation best practice that enables continuous program improvement strategies and supports the centers in meeting goals at their respective institutional level. The RCMI Evaluation Conceptual Model provides a structure to facilitate the close monitoring and careful documentation of RCMI program activities and initiatives to better understand the value-added of this program for individual programs and across RCMI programs [14]. Moreover, establishing standard metrics strengthens the ability of the NIMHD to conduct national evaluation of the RCMI program. Both longstanding and newer centers could benefit from sharing of tools and instruments and finding common questions that site evaluators could adopt. Once instruments/tools are reviewed, a set of questions tied to each of the identified targets could be shared and suggested for use across sites. This would support a more robust national evaluation of the RCMI initiative by the NIMHD.

One of the primary NIMHD RCMI program goals is to foster early stage investigators’ careers, particularly those who focus on health disparity and minority health research. The key metrics identified from this effort should be applied to document productivity and outcomes associated with investigators and early-career faculty who are funded by the RCMI. The RCMI’s Investigator Development Core includes a pilot project program to provide research funding to investigators and foster early research careers. Process metrics are critical to document career advancement of early career investigators and can be used in continuous process improvement. These may include the number and types of workshops attended or feedback via mock reviews. Outcome metrics should include grant submissions/awards and publications among early career investigators funded by their RCMI, including the RCMI Pilot Project programs.

Long-term metrics for documenting the success of the pilot project program should focus on documenting the impact of the RCMI in fostering investigator career progression to becoming an independent researcher, demonstrated by converting pilot or early career funding into efforts to secure research funding on NIH Research Program Project Grants (R01 mechanism) or equivalent grants. Evaluation of how the RCMIs foster investigators’ careers should rely on secondary data sources, whenever possible, to reduce respondent burden. However, the inclusion of primary data sources for selected key metrics ensures investigators’ involvement in the evaluation process and provides a means of demonstrating their individual success. This way, RCMI researchers can serve as “champions” of ongoing evaluation data collection.

Community engagement is a distinctive value-added component of the RCMI programs. As such, evaluation of community engagement is fundamental to supporting the broader goals of RCMI efforts. Assessment of engagement with communities may be measured in various ways, due to the complexity of capturing these activities accurately—i.e., community-specific activities, with little guidance on metrics. However, evaluations should go beyond the current commonly used process metrics (e.g., documentation of memoranda of understanding, use of community engagement principles, or citing the number of community partnerships in funding opportunities). Attempts should be made to document the direction of engagement (community-initiated or investigator-initiated) as well as the level of involvement of communities in all stages of the research process [10]. In cases of RCMI community-engaged research, community partners should be engaged in the decision making process to identify relevant evaluation metrics to ensure that the measures and subsequent results are meaningful to the community [32]. Moreover, evaluating the extent to which RCMIs engage community partners as research collaborators to ensure that communities receive the benefits of the research is fundamental to the mission of the RCMI program and important for continued support by NIMHD.

A culture of evaluation, continuous dissemination of findings to the academic and lay communities, and opportunities for leveraging program and consortium-driven data for program decision making must be created to foster consistent and responsive evaluation tracking. Existing research infrastructure programs, such as NIH’s IDeA Networks of Biomedical Research Excellence [33] and the CTSA [7,29,31], PACHE [19], and National Research Mentoring Network [34] initiatives, may provide guidance on best practices that should be explored.

The EWG’s multiphase mixed-methods approach operationalized and validated the disparate evaluation activities across RCMIs, resulted in an RCMI Evaluation Conceptual Model, and identified metrics and best practices while depicting the diversity of RCMI communities. Meaningful, successful evaluation and tracking are continual processes that facilitate the documentation of impact and should be championed across the institutions [35]. RCMIs should continue to include qualitative methods recognizing that qualitative data is critical to produce results for process and outcome evaluations. Results obtained from interviews, focus groups, observations, and case studies, for example, would help describe and inform impacts of the RCMI program. [36] A key evaluation next step for RCMIs should be to engage a broader collaboration across the programs and include the engagement of NIMHD program leadership and community stakeholders. This effort would establish and designate key, cross-cutting (and unique) RCMI metrics that could demonstrate the successful impact of both long-standing and recent RCMIs.

### 4.2. Limitations

An overall limitation is that not all RCMI evaluators or other key personnel participated in this process, as participation in this effort was voluntary and optional (not a grant or evaluation requirement). Additionally, time constraints and competing demands may not have allowed for survey respondents (evaluators) to consult with their RCMI stakeholders, or respond at all. Having a longer timeline to allow for RCMI evaluators to complete the survey, especially amidst the COVID-19 pandemic, would have been ideal. Furthermore, we did not obtain responses to some survey questions that originally were not phrased as a question. Including pilot testing of our survey items may have caught our inconsistent format of asking for information. Finally, this was not an anonymous survey, and since a Google document was used for collecting information, respondents could see each other’s entries. These limitations might have impacted the accuracy and reliability of responses. The EWG acknowledges that the evaluation metrics presented here may need to be better defined and further refined; however, this was beyond the scope of this manuscript. A working group specifically to critically examine each metric is recommended.

## 5. Conclusions

The process and approach for evaluating the progress of the individual RCMIs and a program-wide evaluation are described in this manuscript, providing original guidance that should be prioritized for RCMI evaluations. Our effort to identify an RCMI evaluation conceptual model, metrics and measures, and best practices and challenges resulted in the development of an integrative pragmatic framework for collecting common critical data points and operationalizing measures and data collection approaches.

Challenges and best practices of evaluating programs are varied and many. The RCMIs are positioned in communities that appreciate a mutually-beneficial research process in an effort to effectively change behaviors and policies that address health disparities in traditionally disenfranchised communities [8,37]. A systematic approach to evaluation must be championed by all program stakeholders and requires the ongoing recording and documenting of the inputs, activities, and outputs to assess key process and outcome metrics. Failure to include the diverse stakeholders and agreed-upon sources of data or information can limit the evaluation team’s ability to accurately identify performance gaps and derive appropriate solutions.

Findings address existing gaps about evaluation approaches for defining metrics and collecting critical data that demonstrate the aggregated impact of the RCMI consortium. This unique and vigorous collaboration of RCMI evaluators and the RCMI Translational Research Network coordinators established key metrics and measures for RCMIs. Collaborative and coordinated data collection, management, analysis, and reporting guided by the RCMI Evaluation Conceptual Model will support each Center in fostering health disparities research, and allow the centers to leverage their collective strength as a consortium to address health disparities. The availability of a guiding evaluation framework and identification of metrics may facilitate more rigorous evaluation approaches and improve the ability of RCMIs to demonstrate the collective impact of their health disparities research programs. Furthermore, obtaining relevant, robust evaluation results will also aid the funding agency, NIMHD, in demonstrating the added value of the RCMI program in achieving the agency’s mission. Ultimately, the impacts of the RCMIs (individually and collectively) will benefit marginalized communities and increase the likelihood of health equity.

## Figures and Tables

**Table 1:** Summary of the key RCMI targets and relevant evaluation metrics identified in this study.

Primary Targets	Secondary Targets	Outcome Metrics (Examples) *
1. Increase Scientific Productivity	Grants	# of grant submissions; # of grant awards; $ amount of grants awarded
	Peer-Reviewed Publications	# of peer-reviewed publications (citing the RCMI); increase in impact factor of peer-reviewed publications
	Scientific dissemination	# of posters/symposia at conferences
	Community dissemination	# of presentations or community events; # of patent applications; # of patent acquisitions; # of studies incorporating community engagement perspectives & partnerships
	Pilot project specific productivity	% (based on total) of pilot projects that secure external funding directly related to the work; % (based on total) of pilot projects that publish (directly related to their pilot work)
2. Increase Scientific Collaborations	Research partners	# of research partners collaborated with (externally); # of research partners collaborated with (internally)
	Community partners	# and type of academic-community partnerships; # and type of community presentations/engagement; # and type of long-term (sustained) community partnerships; # of people added to Community Advisory Boards; # of MOUs signed; # of sectors added to Community Advisory Boards (types of community agencies)
3. Foster Professional Growth	Early Career Investigators	# external competitive grant submissions (clinical or behavioral with a health disparity focus or biomedical); # external competitive grant awards (clinical or behavioral with a health disparity focus or biomedical); # peer-reviewed publications (citing the RCMI); # and type of scientific presentations; Career advancement of RCMI-affiliated (funded) faculty
	Underrepresented Investigators	Grantsmanship training opportunities - # offered and quality; Mentoring quality; # of K- and R-applications submissions; # of K- and R- awards; # of non-NIH applications submitted; # of non-NIH grant awards; # of randomized controlled trial (RCT) applications submitted; # of RCT grant awards
4. Expand Research Resources	Physical infrastructure	Increase in clinical facilities/lab space, offices, cubicles and/or acquisition of equipment/software
	Intellectual resources	# of biostatistics/methodological consultations, workshops, seminars, and/or trainings
	Faculty hires*	# of faculty hires focusing on minority health and disparity research; # of participants that attend conferences organized by RCMI; Online trainings/webinars - # of hits, clicks, and/or views (i.e., reach & engagement); Development of a repository of data, protocols, surveys or other resources

* May not apply to all RCMIs (N/A); #: number; $: USD amount; %: percentage; MOU: memorandum of understanding

**Table 2:** Number of Research Centers in Minority Institutions that completed the different sections of the case study survey (*n* = 14).

Primary Evaluation Target	Outcome Metric Examined	Responses (*n*)
Scientific Productivity	Number of peer-reviewed publications	10
Scientific Collaborations	Number of research partners	9
Professional Growth	Mentoring quality	10
Research Resources	Number of biostatistics consultations, workshops, seminars, and trainings	8
Community Engagement	Number of formal agreements, MOUs, or partnerships with community partners and the RCMI centers	7
Health Disparities	Number of external health disparity focused grants submitted by RCMI funded pilot project investigators	2

**Table 3:** Primary target: Increase scientific productivity. Key findings of the case study regarding the operationalization and data collection strategies for the metric “number of peer-reviewed publications”.

Sub-Questions (N)	Themes	Frequency (%)
How the metric is operationalized (9)	Peer reviewed journals by investigators/studies or affiliated faculty supported by RCMI (cite RCMI)	9 (100)
	Peer reviewed publications that acknowledge/cite RCMI support	6 (66.7)
	Other: Compliance with public access policy; RCMI related (non-peer reviewed) publications	3 (33.3)
Approaches and methods for data collection (10)	Online database (Scopus, Google Scholar, Web of Science, PubMed, PubCrawler, etc.)	8 (80.0)
	Progress report (including NIH RPPR) or survey (RCMI affiliated faculty or facilities)	8 (80.0)
	Other: Tracking system/Administrative records; Bio-sketch/CV; Interviews with supported researchers	7 (70.0)
Data source (9)	Primary and secondary data	9 (100)
Periodicity (9)	Annually	5 (55.6)
	Bi-annually	5 (55.6)
	Other: Ongoing; monthly; or bi-monthly	5 (55.6)

N: Number of RCMIs who responded to each sub-question. The number of respondents varied by question, as all sites did not address all sub-questions.

**Table 4:** Primary Target: Increase scientific collaborations. Key findings of the case study regarding the operationalization and data collection strategies for the metric “number of research partners”.

Sub-Questions (N)	Themes	Frequency (%)
How the metric is operationalized (9)	Collaborator (individual or group: e.g., mentor, Co-I, PI, org., consortium, community group) participating in grant/research related activities	8 (88.9)
	Individual or group who is collaborating with an RCMI funded investigator/study	5 (55.6)
	Community support (partnership building)	4 (44.4)
	Other: Intra/inter-institutional collaboration; Using RCMI facilities with an RCMI co-investigator	6 (66.7)
Approaches and methods for data collection (8)	Survey	4 (50.0)
	Tracking system/administrative records	4 (50.0)
	Progress report	3 (37.5)
	Other: Online database; advisory/steering committee meeting or interview	5 (62.5)
Data source (8)	Primary and secondary data	8 (100)
Periodicity (8)	Ongoing	3 (37.5)
	Bi-annually	3 (37.5)
	Annually	3 (37.5)
	Quarterly	1 (12.5)

N: Number of RCMIs who responded to each sub-question. The number of respondents varied by question, as all sites did not address all sub-questions.

**Table 5:** Primary Target: Foster professional growth of underrepresented investigators. Key findings of the case study regarding the operationalization and data collection strategies for the metric “mentoring quality”.

Sub-Questions (N)	Themes	Frequency (%)
How the metric is operationalized (9)	Perceived (mentoring) quality and satisfaction (reported by mentee and mentor)	7 (88.9)
	Benefit or impact of mentorship	3 (33.3)
	Training needs (mentee)	3 (33.3)
	Other: Early stage investigators participating in RCMI; or promotion & tenure	2 (22.2)
Approaches and methods for data collection (8)	Survey	8 (100)
	Interview	3 (37.5)
	Reports/Plans	2 (25.0)
	Other: Tracking system, proposal information or institutional data	2 (25.0)
Data source (9)	Primary	3 (33.3)
	Primary and secondary	6 (66.7)
Periodicity (9)	Annually	7 (77.8)
	Bi-annually	2 (22.2)

N: Number of RCMIs who responded to each sub-question. The number of respondents varied by question, as all sites did not address all sub-questions.

**Table 6:** Primary target: Expand research resources. Key findings of the case study regarding the operationalization and data collection strategies for the metric “number of biostatistics consults, workshops, seminars, and trainings”.

Sub-Questions (N)	Themes	Frequency (%)
How the metric is operationalized (7)	Training activities (workshops, seminars) offered through RCMI communities	7 (100)
	Support/consultation offered through RCMI communities (biostats etc.)	4 (57.1)
Approaches and methods for data collection (8)	Questionnaire/survey	8 (100)
	Consultation tracking system	4 (50.0)
	Administrative record (for ex., attendance list)	5 (62.5)
	Other: Observation or progress report	2 (25.0)
Data source (8)	Primary	7 (87.5)
	Primary and secondary	1 (12.5)
Periodicity (8)	Ongoing/as per event	7 (87.5)
	Annually	4 (50.0)
	Other: Bi-annually or quarterly	2 (25.0)

N: Number of RCMIs who responded to each sub-question. The number of respondents varied by question, as all sites did not address all sub-questions.

**Table 7:** Primary target: Increase scientific collaborations. Key findings of the case study regarding the operationalization and data collection strategies for the metric “number of partnerships with community partners and the RCMI”.

Sub-Questions (N)	Themes	Frequency (%)
How the metric is operationalized (6)	Community members involved in the RCMI activities and decision making	5 (83.3)
	Community partnership in proposals and research studies supported by RCMI	5 (83.3)
	Member/partner affiliation	3 (50.0)
	Level of involvement	1 (16.7)
Approaches and methods for data collection (7)	Interview or survey	6 (85.7)
	Tracking system/Administrative records	2 (28.6)
	Needs assessment	2 (28.6)
	Other: Progress report; focus group	2 (28.6)
Data source (7)	Primary and secondary	4 (57.1)
	Primary	2 (28.6)
Periodicity (6)	Ongoing	6 (100)

N: Number of RCMIs who responded to each sub-question. The number of respondents varied by question, as all sites did not address all sub-questions.

## Appendix A

**Table A1:** Active research centers in minority institutions (NIH RePORTER, May 2020 ^1^).

**Institutions**	**Support Years**
Florida Agricultural and Mechanical University	35
Ponce School of Medicine	35
Clark Atlanta University	33
Meharry Medical College	33
University of Hawaii at Manoa	33
University of Puerto Rico Medical Sciences	33
Howard University	32
Morehouse School of Medicine	32
Tuskegee University	28
University of Texas El Paso	27
Charles R. Drew University of Medicine & Science	12
Xavier University of Louisiana	12
Florida International University	3
North Carolina Central University	3
Northern Arizona University	3
San Diego State University	2
University of California Riverside	1
Morgan State University	1

^1^ NIH RePORTER search criteria: RFA-MD-17-003, RFA-MD-17-006, RFA-MD-18-012 FY: Active Projects.
